# Schizophrenia Risk Mediated by microRNA Target Genes Overlapped by Genome-Wide Rare Copy Number Variation in 22q11.2 Deletion Syndrome

**DOI:** 10.3389/fgene.2022.812183

**Published:** 2022-04-15

**Authors:** Shengjie Ying, Tracy Heung, Zhaolei Zhang, Ryan K. C. Yuen, Anne S. Bassett

**Affiliations:** ^1^ Institute of Medical Science, University of Toronto, Toronto, ON, Canada; ^2^ Clinical Genetics Research Program, Centre for Addiction and Mental Health, Toronto, ON, Canada; ^3^ The Dalglish Family 22q Clinic, University Health Network, Toronto, ON, Canada; ^4^ Department of Molecular Genetics, University of Toronto, Toronto, ON, Canada; ^5^ Donnelly Centre for Cellular and Biomolecular Research, University of Toronto, Toronto, ON, Canada; ^6^ Department of Computer Science, University of Toronto, Toronto, ON, Canada; ^7^ Department of Psychiatry, University of Toronto, Toronto, ON, Canada; ^8^ Toronto General Hospital Research Institute and Campbell Family Mental Health Research Institute, Toronto, ON, Canada

**Keywords:** schizophrenia, pathway enrichment analysis, neurodevelopmental disorders, DiGeorge syndrome, model system, DGCR8, FMRP, miR-17-5p

## Abstract

The 22q11.2 deletion is associated with >20-fold increased risk for schizophrenia. The presence of gene *DGCR8* in the 22q11.2 deletion region has suggested microRNA (miRNA) dysregulation as possibly contributing to this risk. We therefore investigated the role of miRNA target genes in the context of previously identified genome-wide risk for schizophrenia conveyed by additional copy number variation (CNV) in 22q11.2 deletion syndrome (22q11.2DS). Using a cohort of individuals with 22q11.2DS and documented additional rare CNVs overlapping protein coding genes, we compared those with schizophrenia (*n* = 100) to those with no psychotic illness (*n* = 118), assessing for rare CNVs that overlapped experimentally supported miRNA target genes. We further characterized the contributing miRNA target genes using gene set enrichment analyses and identified the miRNAs most implicated. Consistent with our hypothesis, we found a significantly higher proportion of individuals in the schizophrenia than in the non-psychotic group to have an additional rare CNV that overlapped one or more miRNA target genes (odds ratio = 2.12, *p* = 0.0138). Gene set analyses identified an enrichment of FMRP targets and genes involved in nervous system development and postsynaptic density amongst these miRNA target genes in the schizophrenia group. The miRNAs most implicated included miR-17-5p, miR-34a-5p and miR-124-3p. These results provide initial correlational evidence in support of a possible role for miRNA perturbation involving genes affected by rare genome-wide CNVs in the elevated risk for schizophrenia in 22q11.2DS, consistent with the multi-hit and multi-layered genetic mechanisms implicated in this and other forms of schizophrenia.

## Introduction

The recurrent chromosome 22q11.2 microdeletion that defines 22q11.2 deletion syndrome (22q11.2DS), occurs in an estimated 1 in 2,148 live births and is associated with an over 20-fold increased risk for schizophrenia (McDonald-McGinn et al., 2015; Blagojevic et al., 2021). Amongst mechanisms proposed to confer this schizophrenia risk is microRNA (miRNA) perturbation (Brzustowicz and Bassett, 2012; Forstner et al., 2013; Merico et al., 2014). miRNAs are small regulatory RNA molecules (∼22 nucleotides long) that typically repress translation. The 22q11.2 deletion region contains *DGCR8*, which encodes a critical microprocessor complex in miRNA biogenesis, as well as genes encoding seven miRNAs (Guna et al., 2015). Widespread miRNA perturbance expected due to hemizygosity of these components is supported by miRNA profiling studies in experimental models of the 22q11.2 deletion (Stark et al., 2008; Earls et al., 2012; Zhao et al., 2015; Toyoshima et al., 2016; Chun et al., 2017; Saito et al., 2020). For idiopathic or genetically uncharacterized schizophrenia, miRNA dysregulation has been implicated in studies of post-mortem brain tissue (Beveridge et al., 2010; Moreau et al., 2011; Santarelli et al., 2011) and blood serum (van den Berg et al., 2020), in genome-wide association studies (Ripke et al., 2014; Hauberg et al., 2016), and in miRNA-target gene regulatory network analyses (Guo et al., 2010; Santarelli et al., 2019).

Genome-wide rare copy number variants (CNVs), including but not limited to 22q11.2 deletions, contribute significantly to genetic risk for schizophrenia in the general population (Walsh et al., 2008; Costain et al., 2013; Marshall et al., 2017) and can impart additional risk for schizophrenia in 22q11.2DS (Bassett et al., 2017). Both miRNAs and effects of altered gene dosage related to CNVs can affect gene expression, and their interaction has been explored in both evolutionary (Duan et al., 2009; Felekkis et al., 2011; Marcinkowska et al., 2011) and disease contexts (Schiffman et al., 2011; Willemsen et al., 2011; Qiao et al., 2013; Vaishnavi et al., 2013; Merico et al., 2014; Warnica et al., 2015). Based on the theory that miRNAs can buffer against genetic variation that would otherwise disrupt gene expression (Hornstein and Shomron, 2006), a previous report proposed that miRNA dysregulation imparted by the 22q11.2 deletion may be lowering the tolerance for potentially deleterious variants involving genes for which precise regulation by miRNAs is required for gene function or for maintaining cellular homeostasis (Brzustowicz and Bassett, 2012).

**FIGURE 1:**
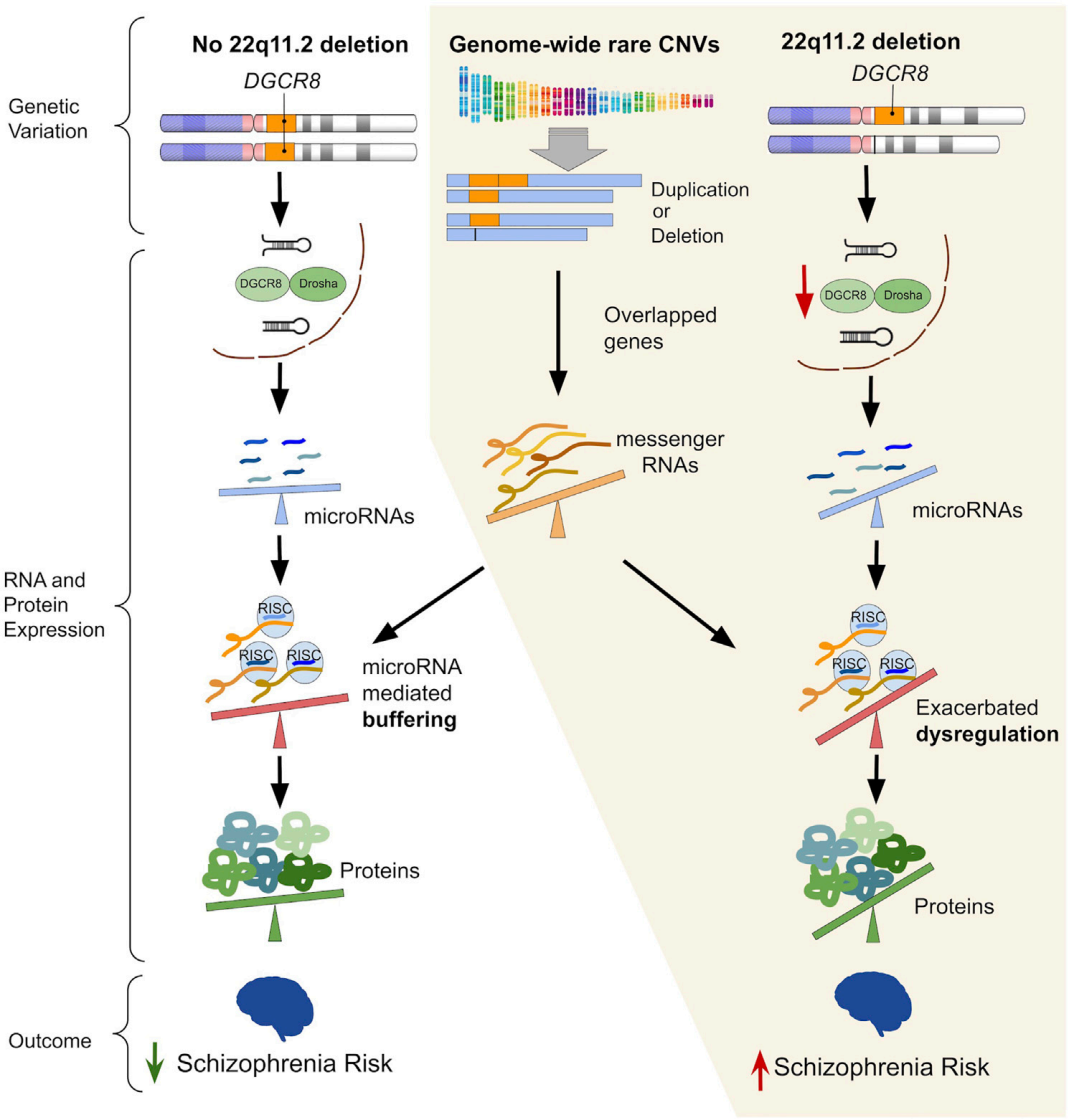
The left-hand column (“No 22q11.2 deletion”) illustrates the ability of an intact microRNA (miRNA) regulatory system to buffer the effects of genetic variation that would otherwise perturb gene expression. The parts grouped by the yellow background illustrate the proposed mechanism of how widespread miRNA dysregulation imparted by the 22q11.2 deletion may exacerbate the downstream impact of rare genome-wide copy number variants (CNVs), thereby increasing risk for schizophrenia. Hemizygosity of *DGCR8* due to the 22q11.2 deletion leads to reduced processing of primary-to pre-miRNAs in miRNA biogenesis, thus resulting in widespread miRNA dysregulation. Subsequently, an altered degree of miRNA targeting of differentially expressed messenger RNA (mRNA) transcripts (due to overlapping rare CNVs) may amplify dysregulation of protein expression. A steeper tilt of the seesaws represents a greater degree of dysregulation (i.e., upregulation or downregulation) of miRNAs, mRNAs, proteins, or miRNA mediated repression/degradation of its target mRNA.

Given the known risk contribution of rare CNVs to schizophrenia expression and the possibility of miRNA effects, we proposed a multi-hit model whereby 22q11.2 deletion-related miRNA perturbance, that could affect genome-wide protein abundance, may exacerbate altered gene-dosage effects of genome-wide rare CNVs (at the level of transcription) (Figure 1). Using the subset of a previously characterized 22q11.2DS cohort with additional (non-22q11.2 deletion) rare CNVs (Bassett et al., 2017), we tested the hypothesis that the individuals with schizophrenia would be more likely to have a miRNA target gene affected by an additional rare CNV, compared to the individuals with no psychotic illness. We also used gene set analyses to examine the putative relevance to schizophrenia of the miRNA target genes involved, and we identified the miRNAs most implicated. To enable this study, we compiled a list of experimentally supported miRNA target genes. We also conducted a review of miRNAs showing evidence of differential expression in available experimental models of the 22q11.2 deletion to provide further context for the findings.

## Materials and Methods

### 22q11.2DS Cohort, Psychiatric Status, and CNV Detection

We used previously published data on additional rare genome-wide CNVs identified in a cohort of 22q11.2DS (Bassett et al., 2017). Details on the original genotyping and phenotyping, and informed consent from all individuals and/or their legal guardian can be found in Bassett et al., 2017. Briefly, among 329 individuals with 22q11.2DS, those diagnosed with schizophrenia or another psychotic illness (e.g., schizoaffective disorder) were assigned to the “schizophrenia group” (*n* = 158) and those with no history of psychotic illness at age ≥25 years were assigned to the “non-psychotic” group (*n* = 171). For this study we identified the subset of individuals (*n* = 218) with one or more autosomal, rare CNVs (additional to the 22q11.2 deletion) overlapping a protein coding gene (i.e., “genic CNVs”) and found no significant differences between groups in the selection of this subset (100 of 158 in schizophrenia vs. 118 of 171 in non-psychotic, *p* = 0.2948, odds ratio (OR) = 0.775). We specifically selected for genic CNVs, defined as overlapping one or more base pairs in an exonic, intronic or untranslated region of a protein coding gene, to encompass all manners in which CNVs may perturb regulation of gene expression (Supplementary Table S1). We defined rare as present in <0.1% of population control databases and all CNVs reported were ≥10 kb in length (Bassett et al., 2017).

As was the case for the whole cohort (*n* = 329) (Bassett et al., 2017), in this subset (*n* = 218) there was a significantly higher proportion of female individuals in the non-psychotic group (*n* = 76; 64%) compared to the schizophrenia group (*n* = 48; 48%) (*p* = 0.020, OR = 1.954 [95% confidence interval (95% CI) = 1.10–3.50].

### miRNA Target Genes

To assess the overlap between rare genome-wide CNVs in the 22q11.2DS cohort and miRNA target genes, we created two sets of miRNA targets: 1) all high-confidence miRNA targets and 2) the subset of genes targeted by a compiled list of miRNAs with evidence of differential expression in 22q11.2 deletion experimental models.

**FIGURE 2:**
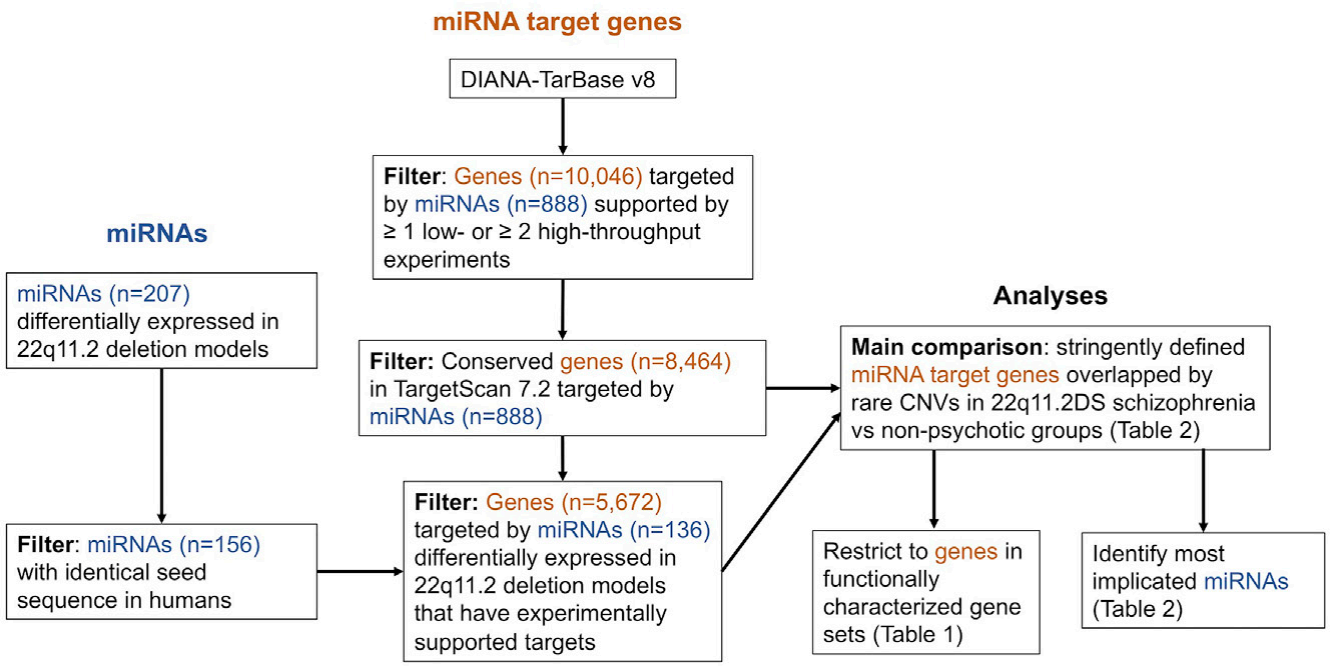
Integrated workflow for the retrieval of microRNAs (miRNAs) with evidence of differential expression in 22q11.2 deletion experimental models, filtering of experimentally supported miRNA target genes from DIANA-TarBase v8 (Karagkouni et al., 2018), and analyses comparing the proportion of individuals between groups with a miRNA target genes overlapped by an additional rare CNV. Each miRNA may target multiple genes and each gene may be targeted by multiple miRNAs.

To obtain a set of all high-confidence miRNA target genes, we opted to use experimentally supported miRNA target genes from DIANA-TarBase v8 (obtained 3 September 2020) (Karagkouni et al., 2018). We used a set of filtering criteria designed to improve stringency, as follows: 1) the positive miRNA-gene interaction is supported by at least one low throughput experiment (e.g., luciferase reporter assay) or two high throughput experiments (e.g., CLIP-seq), 2) the effect on gene expression is listed as downregulated, and 3) the miRNA species is listed as human. We then further restricted these filtered miRNA target genes to genes listed as conserved, human miRNA targets in TargetScan 7.2 (Agarwal et al., 2015). This resulted in 8,464 genes representing high-confidence human miRNA targets (Figure 2, Supplementary Tables S2, S3).

We also created a subset these 8,464 high-confidence target genes by restricting to those targeted by a list of miRNAs we compiled that showed evidence of differential expression in 22q11.2 deletion experimental models (Supplementary Tables S4, S5). This provided a set of 5,672 miRNA targets (Figure 2).

### miRNAs With Evidence of Differential Expression in 22q11.2 Deletion Experimental Models

We compiled a list of miRNAs with evidence of differential expression in all studies of 22q11.2 deletion experimental models with available published data of miRNA profiling in brain or model-brain tissue (Stark et al., 2008; Earls et al., 2012; Zhao et al., 2015; Toyoshima et al., 2016; Chun et al., 2017). These comprised of three studies using mouse models with a 1.2 or 1.3 Mb hemizygous deletion on mouse chromosome 16 (orthologous to the 1.5 Mb deletion on chromosome 22q11.2 in humans) (Stark et al., 2008; Earls et al., 2012; Chun et al., 2017) and two human induced pluripotent stem cell (hiPSC) models derived from individuals with the 3 Mb 22q11.2 deletion (Zhao et al., 2015; Toyoshima et al., 2016) (Supplementary Tables S4, S5).

**FIGURE 3:**
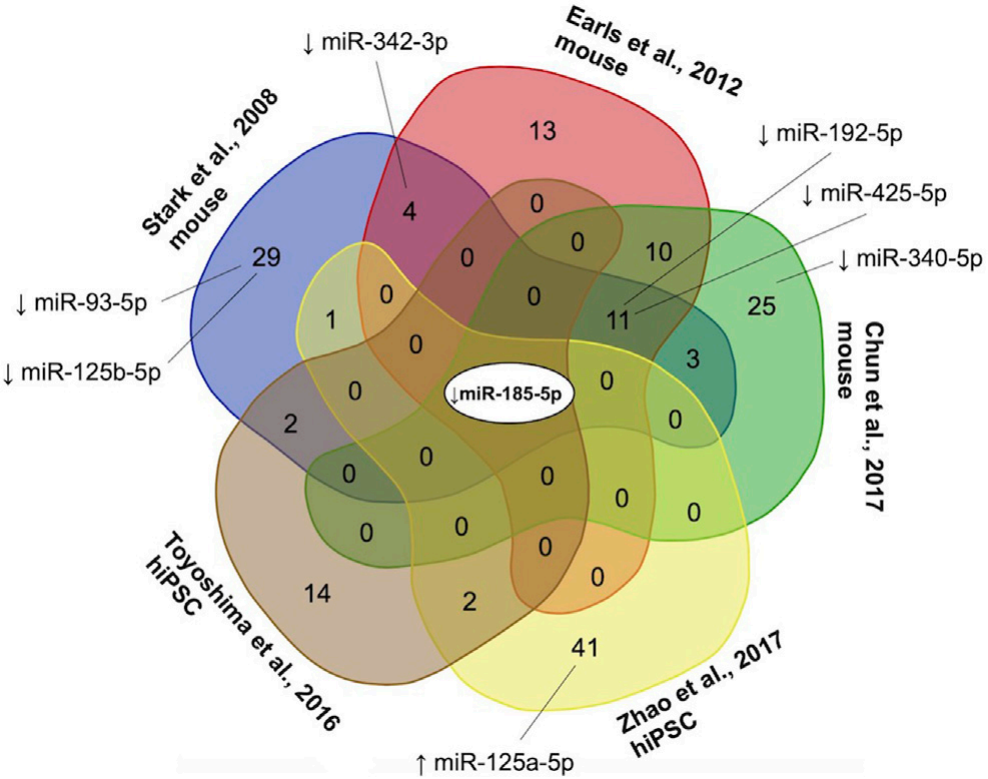
Convergence of microRNAs (miRNAs) reported as differentially expressed in three mouse model studies (Stark et al., 2008; Earls et al., 2012; Chun et al., 2017) and two human induced pluripotent stem cell models (Zhao et al., 2015; Toyoshima et al., 2016) of the 22q11.2 deletion. Collectively, 156 miRNAs were reported as differentially expressed (using various criteria), of which 122 (78.2%) were downregulated, 40 (25.6%) upregulated, and 6 (3.8%) up-or down-regulated in different studies. The only miRNA downregulated in all five studies was miR-185-5p, a conserved miRNA located within the proximal 22q11.2 deletion region (Guna et al., 2015). miRNAs with targets that were found to be overlapped by one or more rare genome-wide CNVs in significantly more individuals with 22q11.2 deletion syndrome in the schizophrenia than non-psychotic group in the current study are shown with a black line connecting to the studies reporting differential miRNA expression. ↑, increased expression; ↓, decreased expression.

To resolve discrepancies in miRNA nomenclature and differences between miRNA species, first all miRNA names were converted to miRBase v21 nomenclature using the R package miRBaseConverter (Xu et al., 2018). If a miRNA named according to a previous nomenclature system mapped onto multiple miRNAs using miRBase v21 nomenclature, all mappings were retained for subsequent analyses. To ensure that miRNAs from mouse models (with the “mmu-” prefix) were applicable for human analyses, we included only mouse miRNAs where the human miRNA (“hsa-” prefix) of the same number listed in miRBase had an identical 6-mer seed sequence (Supplementary Table S5). Mature miRNAs that are different arms (i.e., -5p vs. -3p) of the same precursor (i.e., same number) were considered distinct miRNAs. We used the Van de Peer Lab’s Venn diagram tool^1^ to determine the overlap and generate a multi-group Venn diagram for differentially expressed miRNAs from different studies (Figure 3).

### miRNA Target-CNV Burden, Gene Set Enrichment, and miRNA Analyses

We compared the proportion of individuals between the schizophrenia and non-psychotic groups with one or more miRNA target gene overlapped by an additional rare CNV. This comparison was done first using all high-confidence miRNA targets (n = 8,464) and then restricted to the targets of miRNAs with evidence of differential brain tissue expression in 22q11.2 deletion models (*n* = 5,672) (Table 1; Supplementary Table S6). The proportions of individuals between groups were first compared using a two-sided Fisher’s exact test. Additionally, we created a logistic regression model for schizophrenia expression that included the following variables: the presence of a miRNA target gene overlapped by an additional rare genome-wide CNV, the summed size in base pairs of all rare CNVs (excluding the 22q11.2 deletion) per individual, the number of protein coding genes overlapped by all rare CNVs (excluding the 22q11.2 deletion) per individual, and sex.

**TABLE 1:** Schizophrenia expression in 22q11.2DS and its relationship to having a miRNA target gene overlapped by an additional rare CNV.

	Individuals with an additional genome-wide rare genic CNV overlapping a miRNA target gene				Unadjusted analysis				Adjusted analysis^a^			
	Schizophrenia (n = 100)		Non-psychotic (n = 118)									
	n	%	n	%	OR	95% CI		*p*	OR	95% CI		*p*
All miRNA target genes (n = 8,464)^b^												
All Rare CNVs^c^	69	69.0	58	49.2	2.29	1.27	4.18	0.0038	2.12	1.17	3.85	0.0138
Gene Set Restriction^d^												
FMRP targets Darnell^e^	11	11.0	4	3.4	3.50	1.00	15.59	0.0327	3.45	1.03	11.62	0.0455
FMRP targets Ascano^e^	16	16.0	8	6.8	2.61	1.00	7.39	0.0488	2.73	1.03	7.26	0.0437
Nervous system development^e^	23	23.0	13	11.0	2.40	1.09	5.51	0.0271	1.85	0.84	4.10	0.1273
Neuro-function union inclusive^e^	28	28.0	18	15.3	2.15	1.06	4.47	0.0298	1.65	0.81	3.37	0.1661
Brain expression high/medium^f^	42	42.0	31	26.3	2.03	1.10	3.75	0.0151	1.84	0.99	1.22	0.0555
Endocrine/exocrine/reproduction^g^	20	20.0	10	8.5	2.69	1.13	6.80	0.0175	2.55	1.09	1.45	0.0311
22q11.2 deletion models miRNA targets subset (n = 5,672)^h^												
All Rare CNVs^c^	58	58.0	49	41.5	1.82	1.02	3.25	0.0398	1.64	0.91	2.97	0.097

CNV, copy number variant; Unadjusted analysis, two-sided Fisher’s exact test; OR, odds ratio; 95% CI, 95% confidence interval of the odds ratio; miRNA, microRNA; FRMP, Fragile X Mental Retardation Protein.

a The *p*-values, ORs, and 95% CIs listed are for the presence of a miRNA target overlapped by a rare CNV in a logistic regression model with the following additional variables: the total rare CNV size per individual, total number of protein coding genes overlapped by a rare CNV per individual, and sex.

b miRNA, target genes retrieved from DIANA-TarBase v8, supported by experimental evidence (see Methods for filtering criteria).

c Rare CNVs, with <0.1% frequency in control databases and ≥10 kb in length.

d Restricting to individuals with a rare CNV overlapping a miRNA target gene that belongs to a gene set.

e Among 19 gene sets belonging to Neuro-functional category of gene sets (Supplementary Table S7). The larger Neuro-function union inclusive gene set includes all genes from the Nervous system development gene set (Supplementary Table S9).

f Among seven gene sets belonging to BrainSpan category of gene sets (Supplementary Table S7).

g Among seven gene sets belonging to mouse organ systems category of gene sets (Supplementary Table S7).

h Subset of the 8,464 genes that are targets of miRNAs, that were found to be differentially expressed in experimental models of the 22q11.2 deletion (Figure 3).

To assess the representation of miRNA target genes overlapped by CNVs with respect to gene function related to neurodevelopmental pathways, brain expression, and other organ systems, we used 36 gene sets from a large study of rare CNVs in idiopathic schizophrenia (Marshall et al., 2017) (Supplementary Table S7). We used two approaches for gene set enrichment analyses to accommodate for the potential biases of each method (see Supplementary Methods for detailed explanation). Briefly, we first applied a “self-contained” method by comparing the proportion of individuals between groups with a rare CNV overlapping a miRNA target gene that also belongs to a gene set, using a two-sided Fisher’s exact test and the previously described logistic regression model. Second, using a “competitive” approach, we divided the genes overlapped by CNVs in the schizophrenia and non-psychotic groups by those that were or were not deemed stringent miRNA targets genes, resulting in four subgroups: 1) miRNA targets and 2) non-miRNA targets from the schizophrenia group, and 3) miRNA targets and 4) non-miRNAs targets from the non-psychotic group. For each subgroup we used a one-sided Fisher’s exact test (i.e., conventional hypergeometric test for enrichment) (Reimand et al., 2019) to compare the proportion of genes in the subgroup versus all other genes in the genome that belong to a particular gene set, using 19,203 distinct Ensembl IDs of protein coding genes with from HGNC^2^ as the background. The self-contained approach measures the relative degree of enrichment between the schizophrenia and non-psychotic groups and is resistant to findings that may be driven by an anonymously large number of genes being overlapped by CNVs in small number of individuals, which may occur when using the competitive method. In contrast, the competitive method measures enrichment relative to the background genome and is resistant to findings driven by the same (or a small number of genes) repeatedly being overlapped by CNVs in an anonymously large number of individuals, which may occur when using the self-contained method. Benjamini–Hochberg false discovery rate (FDR) was used to adjust for multiple testing separately for each of these gene set categories: neuro-functional (*n* = 19), mouse-neuro (*n* = 3), brain expression (*n* = 7), and mouse body systems (*n* = 7).

To determine which miRNAs were the ones whose target genes contributed most to the differential burden of miRNA targets overlapped by CNVs between the schizophrenia and non-psychotic groups, we defined two categories of miRNAs. The first are “broadly targeting” miRNAs which we defined as miRNAs that contributed to the results of one or more individuals in both groups. The second are “exclusively targeting” miRNAs which contributed to the results of only one group. For each category, we determined the miRNAs that contributed to the results of significantly more individuals in one group compared to the other. Using a two-sided Fisher’s exact test, with the threshold of *p* < 0.05, we determined *a priori* that a miRNA deemed exclusively targeting would need to contribute to the results of either 4 of 100 individuals in the schizophrenia group (versus 0 of 118 in non-psychotic) or 6 of 118 individuals in the non-psychotic group (versus 0 of 100 in schizophrenia) to reach significance (Table 2).

**TABLE 2:** miRNAs with target genes overlapped by rare CNVs in significantly more individuals in the 22q11.2DS schizophrenia group than in the 22q11.2DS non-psychotic group.

		Individuals with rare genic CNV overlapping a miRNA target^a^				Analysis			Evidence of miRNA dysregulation in	
		Schizophrenia (n = 100)		Non-psychotic (n = 118)						
Target genes of miRNA overlapped by CNVs in	miRNA (hsa-miR-)	n	%	n	%	OR	*p*	FDR^b^	22q11.2 deletion experimental models^c^	Idiopathic schizophrenia and other neuropsychiatric disorders
1) ≥1 individuals in both the schizophrenia and non-psychotic groups (i.e. “broadly targeting”)^d^	17-5p	15	15.0	5	4.2	3.96	0.0085	0.6747	17-**3p** ↓ hiPSC Toyoshima et al. (2016)	- ↑ expression in schizophrenia post-mortem brain tissue (cortex) Beveridge et al. (2010); Santarelli et al. (2011); Wong et al. (2013)
										- ↓ circulating levels in schizophrenia Shi et al. (2012)
	124-3p	18	18.0	8	6.8	3.00	0.0123	0.6747	None	- ↑ circulating levels in major depression Roy et al. (2017)
										- Essential role in neural precursor differentiation Fineberg et al. (2009)
	34a-5p	18	18.0	9	7.6	2.65	0.0238	0.6747	34**b-3p** & 34**c**-5p ↑ hiPSC Zhao et al. (2015)	- ↑circulating levels in schizophrenia and dysregulated in major depression, bipolar, Alzheimer’s, and Parkinson’s Lai et al. (2011); van den Berg et al. (2020)
										- ↑circulating levels in early onset schizophrenia Chen et al. (2021)
	128-3p	7	7.0	1	0.8	8.73	0.0253	0.6747	None	-Involved in neural migration, excitability, apoptosis, development, and has been implicated in schizophrenia, intellectual disability, epilepsy, Alzheimer’s, and motor dysfunction Tan et al. (2013); Ching and Ahmad-Annuar (2015); Franzoni et al. (2015); Zhou et al. (2018); Shu et al. (2019)
										-Regulates astroglia receptor signalling in an FMRP dependent manner Men et al. (2020)
	20b-5p	7	7.0	1	0.8	8.73	0.0253	0.6747	None	- Aggravates apoptosis in Alzheimer’s mouse model Tian et al. (2021)
	93-5p	13	13.0	5	4.2	3.36	0.0253	0.6747	↓ mouse Stark et al. (2008)	- Target gene overlapped by *de novo* CNV in intellectual disability Qiao et al. (2013)
										- Ortholog of the miR-17/92 cluster Mogilyansky and Rigoutsos (2013)
	192-5p	6	6.0	1	0.8	7.41	0.0490	1.0000	↓ mouse Stark et al. (2008); Earls et al. (2012); Chun et al. (2017)	- Upregulation rescues cognitive impairment in a depression mouse model Tang et al. (2019)
2) Only the schizophrenia group (i.e., “exclusively targeting”)^e^	342-3p	5	5.0	0	0	Inf	0.0192	0.0428	↓ mouse Stark et al. (2008); Earls et al. (2012)	- Circulating miR-342-5p↓ in schizophrenia Gardiner et al. (2012)
	125a-5p	4	4.0	0	0	Inf	0.0428	0.0428	↑ hiPSC Zhao et al. (2015)	- Circulating miR-125a-3p & miR-125b-3p ↑ in schizophrenia Camkurt et al. (2016)
	125b-5p	4	4.0	0	0	Inf	0.0428	0.0428	↓ mouse Stark et al. (2008)	-↑ expression in schizophrenia post-mortem brain tissue (cortex) Beveridge et al. (2010)
										- Interacts with FMRP in mouse brain to affect synaptic plasticity Edbauer et al. (2010)
	340-5p	4	4.0	0	0	Inf	0.0428	0.0428	↓ mouse Chun et al. (2017)	- ↑ expression in schizophrenia post-mortem brain tissue (cortex) Beveridge et al. (2010)
										-↑ expression in FMRP knock out mice Liu et al. (2015)
	425-5p	4	4.0	0	0	Inf	0.0428	0.0428	↓ mouse Stark et al. (2008); Earls et al. (2012); Chun et al. (2017)	- Dysregulated expression in schizophrenia post-mortem brain tissue (cortex) Moreau et al. (2011); Santarelli et al. (2011)

CNV, copy number variant; Analysis, two-sided Fisher’s exact test; miRNA, microRNA; FDR, Benjamini-Hochberg false discovery rate; ↑, increased expression; ↓, decreased expression.

a Number of individuals in each group with one or more genes targeted by the miRNA of interest and overlapped by an additional rare genome-wide CNV.

b Calculated separately for each set of miRNAs satisfying the criteria for the “broadly targeting” (n = 160) and “exclusively targeting” (n = 5) criterion.

c Indicates if the miRNA of interest or a closely related form is in the list compiled in this study of miRNAs with evidence of differential expression in 22q11.2 deletion models. Differences in closely related forms are bolded.

d Between group comparisons were made for miRNAs that targeted genes overlapped by rare CNVs in ≥1 individual(s) in both groups. One hundred sixty miRNAs satisfied this criterion and the seven that reached significance (*p* < 0.05) are shown.

e Between group comparisons were made for miRNAs that targeted genes overlapped by one or more rare CNVs in ≥4 individuals in the schizophrenia group and none in the non-psychotic group (*p* < 0.05).

All statistical analyses were performed using R 4.0.3. Statistical significance was defined as *p* < 0.05. This study used entirely previously published or open-source data (Stark et al., 2008; Earls et al., 2012; Agarwal et al., 2015; Zhao et al., 2015; Toyoshima et al., 2016; Bassett et al., 2017; Chun et al., 2017; Marshall et al., 2017; Karagkouni et al., 2018).

## Results

### Increased Burden of miRNA Target Genes Overlapped by Rare CNVs in 22q11.2DS Schizophrenia

Using the list of all high-confidence miRNA target genes (*n* = 8,464), consistent with our hypothesis, we found a significantly higher proportion of individuals in the schizophrenia group compared to the non-psychotic group to have one or more miRNA target genes overlapped by an additional rare genome-wide CNV (69 of 100 vs. 58 of 118; *p* = 0.0038; OR = 2.29 [95% CI = 1.47–4.18]) (Table 1). Presence of a miRNA target gene overlapped by a rare CNV remained a significant predictor of schizophrenia expression in a multivariable logistic regression model that adjusted for total rare CNV size, total number of genes overlapped by a rare CNV, and sex (*p* = 0.0138, OR = 2.12 [95% CI = 1.17–3.85]). Male sex was also a significant covariate (*p* = 0.0125). When considering rare duplications or deletions separately, the differential burden of miRNA target genes overlapped by CNVs between groups trended in the same direction, but did not reach statistical significance for either dosage subgroup (Supplementary Table S6).

We tested the effect of this miRNA target gene-CNV burden if we restricted to gene targets of the miRNAs that showed differential expression compared to controls identified in our review of available experimental models (*n* = 5) of the 22q11.2 deletion (Stark et al., 2008; Earls et al., 2012; Zhao et al., 2015; Toyoshima et al., 2016; Chun et al., 2017). Of the 156 miRNAs with available evidence of differential expression, convergence was low and most (*n* = 122, 78.2%) were reported in only a single study (Figure 3). Proceeding with the 136 miRNAs with experimentally supported target genes (*n* = 5,672, Figure 3, Supplementary Tables S4, S5), and rerunning analyses revealed more modest effect size for miRNA targets overlapped by a rare CNV as a predictor of schizophrenia status, and results no longer reached significance when adjusted for other variables in the logistic regression model (Table 1). We therefore used all high-confidence miRNA target genes (*n* = 8,464) for subsequent analyses.

### Gene Set Enrichment Analyses

We assessed for gene set enrichment using two approaches. First, we used a “self-contained” approach by comparing the proportion of individuals between the schizophrenia and non-psychotic groups with an additional rare CNV overlapping a miRNA target gene that also belongs to a gene set. Second, we used a “competitive” approach that pools the genes overlapped by CNVs into four subgroups (divided by schizophrenia versus non-psychotic status, and whether the gene is or is not a miRNA target), and tests for enrichment compared to all other genes in the genome (i.e., “background”).

Using the self-contained approach, six gene sets were nominally enriched in the schizophrenia group (*p* < 0.05) (Table 1, Supplementary Tables S8, S9). Among the four in the neuro-functional category, only the two gene sets representing targets of the Fragile X Mental Retardation Protein (FMRP) (Darnell et al., 2011; Ascano et al., 2012) remained significant after adjusting for other variables using the previously described logistic regression model. We noted that 23 of the 29 genes in the neuro-functional union inclusive gene set contributing to these results are from the nervous system development gene set (Supplementary Table S9), demonstrating the nervous system development set as the main driver of the enrichment observed in the union set. Among the other two gene set categories, we observed enrichment in the schizophrenia group for genes with high/medium expression in the brain and with endocrine/exocrine/reproductive involvement. After correcting for multiple testing (FDR <0.05), no gene sets using the self-contained approach reached significance.

Using the competitive approach, we observed convergence with the self-contained approach in enrichment, after correcting for multiple testing (FDR <0.05), among miRNA target genes overlapped by rare CNVs in the schizophrenia group for the FMRP targets Ascano (OR = 3.51, FDR = 1.27 × 10^–4^) and the nervous system development (OR = 1.93, FDR = 0.0331) gene sets (Supplementary Table S10). We also found that in both the schizophrenia and non-psychotic groups, miRNA targets overlapped by rare CNVs were enriched for genes with high/medium, and prenatal expression in the brain. However, the self-contained approach demonstrated that the enrichment for high/medium brain expression is relatively greater in the schizophrenia group (Table 1; Supplementary Table S10). In contrast, non-miRNA target genes were enriched in both schizophrenia and non-psychotic groups for genes with minimum brain expression (FDR <0.05). Lastly, we found two gene sets, post synaptic density genes (OR = 2.24, FDR = 0.0171) and genes with both pre- and post-natal brain expression (OR = 1.61, FDR = 0.0391) to be enriched only among miRNA targets in the schizophrenia group (Supplementary Table S10). However, these two gene sets did not reach nominal significance using the self-contained approach (Supplementary Table S8).

### miRNAs That Target Genes Overlapped by Rare CNVs in 22q11.2DS

We subsequently performed an exploratory analysis to determine which individual miRNAs contributed most to the differential CNV-target gene burden observed (Table 2; Supplementary Table S11). Among “broadly targeting” miRNAs (defined as contributing to the results of at least one individual in both groups), seven miRNAs (miR-17-5p, miR-124-3p, miR-34a-5p, miR-128-3p, miR-20b-5p, miR-93-5p, and miR-192-5p) had target genes overlapped by CNVs in significantly more individuals in the 22q11.2DS schizophrenia than the non-psychotic group (Table 2). No miRNAs deemed broadly targeting contributed to the results of significantly more individuals in the non-psychotic group compared to the schizophrenia group (Supplementary Table S11). Additionally, among “exclusively targeting” miRNAs (defined as contributing to the results of only one group), there were five miRNAs (miR-342-3p, miR-125a-5p, miR-125b-5p, miR-340-5p, and miR-425-5p) that targeted genes overlapped by rare CNVs exclusively in the schizophrenia group (*p* < 0.05) (Table 2) while no miRNAs contributing exclusively to the non-psychotic group reached significance (Supplementary Table S11).

When we examined the targets of the only miRNA reported as downregulated in all experimental models of the 22q11.2 deletion, miR-185-5p (located in the 22q11.2 deletion region) (Figure 3), that were overlapped by rare CNVs, we found no differential burden between the schizophrenia and non-psychotic groups (3 of 100 vs. 3 of 118; *p* = 1.0000, OR = 1.18 [95% CI = 0.15–9.05]).

## Discussion

In this study, we explore a theoretical proposition that additional rare genome-wide CNVs overlapping genes targeted by miRNAs may contribute to risk for schizophrenia in individuals with a 22q11.2 deletion—the largest known molecular genetic risk contributor for schizophrenia (Bassett et al., 2017; Cleynen et al., 2020). Using a well-characterized 22q11.2DS cohort and stringently defined miRNA targets, we provide correlational evidence in support of our hypothesis by demonstrating that in individuals with a 22q11.2 deletion and an additional rare genic CNV, a significantly higher proportion of those in the schizophrenia group than those with no psychotic illness had one or more miRNA target genes overlapped by an additional rare CNV. This difference remained significant after correcting for the size and genic content of the CNVs. Results of gene set enrichment and selected miRNA analyses support the possibility that dysregulation involving both altered gene dosage and miRNA-related effects may differentially impact schizophrenia-relevant pathways, including genes with high expression in the brain. Although limited by sample size and the reliability/reproducibility of available miRNA data, the results support our proposed model. This model and the evidence available contribute novel insights into possible mechanisms behind how miRNA perturbance, that is likely to be amplified by the 22q11.2 deletion, together with genome-wide variation, may increase risk for schizophrenia in 22q11.2DS, as part of the polygenic contributors to this complex disease.

### Genetic Contributors to Expression of Schizophrenia in 22q11.2DS

Previous evidence supports genome-wide variation in modifying the risk imparted by the 22q11.2 deletion (Bassett et al., 2017; Cleynen et al., 2020). These findings for 22q11.2DS are consistent with those in idiopathic schizophrenia of an enrichment of genome-wide rare CNVs overlapping both protein coding genes (Costain et al., 2013; Marshall et al., 2017) and miRNA coding sequences (Warnica et al., 2015), as well as association with common variants mostly residing in non-coding regions (Ripke et al., 2014). Together, the evidence suggests that multiple mechanisms likely contribute to the dysregulation of gene expression in the etiology of schizophrenia.

Results of the present study suggest an additional layer to how rare genome-wide CNVs overlapping a broad category of genes—those with evidence of being targeted by miRNAs—may contribute to schizophrenia risk in 22q11.2DS. This finding is consistent with both the elevated miRNA dysregulation (Stark et al., 2008; Earls et al., 2012; Zhao et al., 2015; Toyoshima et al., 2016; Chun et al., 2017) and increased penetrance of schizophrenia (Bassett and Chow, 2008; Marshall et al., 2017) in 22q11.2DS. Widespread miRNA dysregulation attributable to *DGCR8* hemizygosity may serve as part of the “threshold lowering” mechanism of the 22q11.2 deletion for schizophrenia risk. Results of the current study are consistent with our proposed theory that part of this lowered threshold may be mediated by miRNA dysregulation exacerbating the pathogenicity of additional rare CNVs in individuals where such CNVs overlap a miRNA target gene (Figure 1).

The results also support miRNA and CNV mediated dysregulation of gene expression differentially impacting genes in schizophrenia-relevant pathways. Consistent with studies of idiopathic schizophrenia (Clifton et al., 2020), the results of gene set analyses in the current study indicate that miRNA target genes overlapped by rare CNVs are enriched for genes that are also targets of FMRP in individuals with 22q11.2DS-schizophrenia (Table 1; Supplementary Tables S8, S10). FMRP, like miRNAs, primarily functions to suppress translation (Laggerbauer et al., 2001; Li et al., 2001; Napoli et al., 2008; Darnell et al., 2011; Ascano et al., 2012) and may act, in some cases, in a miRNA-dependent manner (Jin et al., 2004; Edbauer et al., 2010; Muddashetty et al., 2011; Liu et al., 2015). It is conceivable that miRNA dysregulation may alter the ability of FMRP to regulate a shared target gene, adding yet another layer of perturbation. Notably, the previous analysis of rare CNVs in this cohort did not identify an enrichment of the FMRP gene sets in schizophrenia (Bassett et al., 2017), demonstrating the added utility of the focus on miRNA target genes. Additionally, we found an enrichment in miRNA target genes overlapped by rare CNVs in the schizophrenia group for genes involved in nervous system development (Table 1; Supplementary Table S8, S10). This result is consistent with perturbations in gene expression in early development posited by the neurodevelopmental model of schizophrenia (Birnbaum and Weinberger, 2017) and the established roles of miRNAs in neurodevelopment (Fineberg et al., 2009).

Further supporting the involvement of miRNA dysregulation in this proposed mechanism, there is independent evidence for dysregulation in schizophrenia and/or other neuropsychiatric disorders of the miRNAs most implicated in the findings in this study (Table 2). Amongst these miRNAs are miR-125b, miR-340, and miR-128-3p, which have been demonstrated in mouse functional studies to interact with FMRP (Muddashetty et al., 2011; Liu et al., 2015; Men et al., 2020). The absence of miR-185-5p amongst these miRNAs most implicated suggests that miRNA perturbance imparted by *DGCR8* hemizygosity may affect pathways that are beyond the downstream targets of miR-185-5p (Xu et al., 2013).

### Advantages and Limitations

We used the largest cohort of 22q11.2DS, with and without schizophrenia, with publicly available data on additional rare CNVs (Bassett et al., 2017). The advantages of the sampling strategy and rigorous CNV analytic methods used have been previously described (Bassett et al., 2017). Nonetheless, the cohort is small compared to several other genetic studies of schizophrenia that do not focus on a rare genetic model of schizophrenia (Marshall et al., 2017). The cohort size limited power, particularly for secondary analyses that required restricting to smaller subsets (e.g., evaluating duplications and deletions separately, gene set analyses involving multiple testing).

A challenge faced by all studies that involve miRNA targets is developing a set of accurate miRNA-target gene interactions based on currently available prediction algorithms and experimental databases (Riolo et al., 2021). Due to the high false positive rate of bioinformatically driven miRNA target prediction programs (Pinzón et al., 2017), we opted to rely primarily on experimentally supported miRNA targets (Karagkouni et al., 2018) with additional filtering criteria for added stringency. However, experimentally supported targets are likely biased towards more intensively studied miRNAs (most often those with lower miR-numbers) and genes (e.g., cancer-related *PTEN* and *NF1*).

A combination of factors may be involved in the stronger signal observed when using all miRNA target genes than when restricting to the targets of differentially expressed miRNAs derived from available studies of 22q11.2 deletion experimental models (Table 1). We opted to use brain derived profiling for relevance to schizophrenia. Using other tissues (e.g., circulating miRNAs from blood serum (de la Morena et al., 2013; Sellier et al., 2014)) may produce different results. Our review of these model-derived differentially expressed miRNAs showed little convergence across studies (Figure 3; Supplementary Table S4, S5). Low reproducibility is common in miRNA profiling studies (Baker, 2010; Wang et al., 2011; Chugh and Dittmer, 2012; Witwer and Halushka, 2016) and differences in methodology and other biological factors may have contributed to discordant findings (e.g., statistical criteria, miRNA profiling technologies, mouse models versus hiPSCs, differing 22q11.2 deletion extents, and developmental timing) (Supplementary Table S4). There is also the possibility that because miRNA expression is temporally regulated across developmental stages (Sempere et al., 2003; Caygill and Johnston, 2008), finding miRNAs that were uniquely differentially expressed in each study may imply that the scope of 22q11.2 deletion-related miRNA dysregulation across all developmental stages is broader than what is reported in any individual study, and that currently available results from model systems may underestimate the entirety of miRNA dysregulation effects in 22q11.2DS.

In summary, within the limitations of the sample size and miRNA source data as discussed above, the results of this study offer correlational evidence to suggest that genes targeted by miRNAs and overlapped by additional rare genome-wide CNVs may be conferring part of the risk for schizophrenia in individuals with a 22q11.2 deletion. We postulate that this effect is mediated by widespread miRNA dysregulation imparted by the 22q11.2 deletion through hemizygosity of *DGCR8*. Our proposed theory supports a model of schizophrenia risk that involves multiple layers of perturbance across many genes, pathways, and mechanisms, consistent with the complex genetic architecture of schizophrenia. This study would benefit from being replicated to test for reproducibility as larger cohorts of individuals with 22q11.2DS and CNV data become available, and as miRNA profiling studies and target gene databases/prediction programs improve in their degree of reliability. Additionally, future studies of 22q11.2DS may consider investigating other rare variant types impacting miRNA target genes. It would also be valuable to assess if this category of genes, or other genetic mechanisms involving miRNA dysregulation, also increases risk for schizophrenia in the general population.

## Data Availability

The original contributions presented in the study are included in the article/Supplementary Material, further inquiries can be directed to the corresponding author.

## Footnotes

1
http://bioinformatics.psb.ugent.be/webtools/Venn/.

2
https://www.genenames.org/download/statistics-and-files/.
