# Supplementary material for: Schizophrenia Risk Mediated by microRNA Target Genes Overlapped by Genome-Wide Rare Copy Number Variation in 22q11.2 Deletion Syndrome
Source: Front Genet. 2022 Apr 15;13:812183. doi: 10.3389/fgene.2022.812183 (PMC9053669; doi:10.3389/fgene.2022.812183)
Supplement: Supplementary file 1 [file DataSheet1.docx]

**Supplementary Methods**

***Gene set enrichment analyses***

Two gene set enrichment analysis approaches were used in this study. 1) The “self-contained” approach compares the proportion of individuals with functionally characterized genes overlapped by rare CNVs between the schizophrenia and non-psychotic groups. 2) The “competitive” approach tests for an over-representation of genes compared to the rest of the genome in a certain gene set when pooling together genes overlapped by CNVs from a group of individuals. We used these two methods to guard against enrichments that may appear in one of the two methods driven by potential extreme distributions of genes across CNVs or individuals (e.g., a large number of individuals in one group having a CNV overlapping the same gene that belongs to a gene set or one individual having a CNV overlapping a large number of genes that all belong to a gene set).

*1. Self-contained gene set analysis (Table 1, Supplementary Table S8):*

This method compared, as a proportion of all individuals with a rare genic CNV, individuals that satisfied the following criteria:

- Has an additional rare genome-wide CNV overlapping a miRNA target gene
- The miRNA target gene overlapped by the rare CNV is also in a gene set (Supplementary Table S7)

A two-sided fisher’s exact test and the same logistic regression formula used for the main comparison was used to compare proportions of schizophrenia and non-psychotic individuals.

*2) Competitive gene set analysis (Supplementary Table S10):*

Genes overlapped by rare genome-wide CNVs in this cohort were pooled into four categories:

1. miRNA target genes in schizophrenia individuals (i.e., “SZ_miRNATargets”)
2. Genes that are not miRNA targets in schizophrenia individuals (i.e., “SZ_non-miRNATargets”)
3. miRNA target genes in non-psychotic individuals (i.e., “NP_miRNATargets”)
4. Genes that are not miRNA targets in non-psychotic individuals (i.e., “NP_non-miRNATargets”)

Enrichment was calculated using a one-sided Fisher’s exact test by using the following contingency table (i.e., conventional hypergeometric test for enrichment). The example shown uses the SZ_miRNATargets subgroup (contains 134 CNV-overlapped genes pooled together from 69 individuals), FMR1_Targets_Ascano gene set (n=927), and 19,203 protein coding genes listed in HGNC as the background genome.

|  | Subgroup of genes overlapped by rare CNVs | Not in subgroup of genes overlapped by rare CNVs (i.e., rest of the genome) |
| --- | --- | --- |
| In gene set | **20** | 927 – 20 = **907** |
| Not in gene set | 134 – 20 = **114** | 19,203 – 907 – 20 – 114 = **18,162** |

R output:

Fisher's Exact Test for Count Data

data: matrix(c(20, 114, 907, 18162), nrow = 2)

p-value = 6.695e-06

alternative hypothesis: true odds ratio is greater than 1

95 percent confidence interval:

2.243953 Inf

sample estimates:

odds ratio

3.512606

*Multiple testing adjustment*

Gene sets were grouped into the following four categories: Neuro-functional (n=19), brain expression (n=7), mouse-neuro (n=3), and mouse organ systems (n=7). Benjamini-Hochberg false discovery rate was calculated separately for each category of gene sets, for both gene set analysis methods.
